# Peptide-based PROTAC degrader of FOXM1 suppresses cancer and decreases GLUT1 and PD-L1 expression

**DOI:** 10.1186/s13046-022-02483-2

**Published:** 2022-09-29

**Authors:** Kun Wang, Xiaoyong Dai, Albert Yu, Chunyan Feng, Kewei Liu, Laiqiang Huang

**Affiliations:** 1 Shenzhen Key Laboratory of Gene and Antibody Therapy, Center for Biotechnology and Biomedicine, State Key Laboratory of Chemical Oncogenomics, State Key Laboratory of Health Sciences and Technology, Shenzhen, China; 2 Precision Medicine and Healthcare Research Center, Tsinghua-Berkeley Shenzhen Institute (TBSI), Shenzhen, China; 3 Institute of Biopharmaceutical and Health Engineering, Shenzhen International Graduate School, Tsinghua University, Shenzhen, 518055 Guangdong China; 4 School of Life Sciences, Tsinghua University, Beijing, 100084 China

**Keywords:** P-PROTACs, FOXM1, Liver cancer, Breast cancer, GLUT1, PD-L1

## Abstract

**Background:**

Peptide proteolysis-targeting chimeras (p-PROTACs) with advantages of high specificity and low toxicity have emerged as a powerful technology of targeted protein degradation for biomedical applications. FOXM1, a proliferation-associated transcription factor, is overexpressed in a variety of human tumors as a key driver of tumorigenesis and cancer progression, and is a potential anticancer therapeutic target. However, FOXM1-targeting p-PROTACs has not been researched.

**Methods:**

Here, we first analyzed the expression of FOXM1, GLUT1 and PD-L1 in liver cancer through database and clinical samples of patients. FOXM1-targeting peptides, selected by screening phage display library, are verified its targeting effect by immunofluorescence and CCK-8 test. The novel p-PROTAC degrader of FOXM1 is chemically synthesis, named FOXM1-PROTAC, by linking a FOXM1-binding antagonistic peptide, with the E3 ubiquitin ligase recruitment ligand Pomalidomide and with the cell membrane penetrating peptide TAT. Its degradation effect on FOXM1 was detected by Western blotting, qPCR, and we verified its effect on the behavior of cancer cells by flow cytometry, scratch assay, and Transwell in vitro. The tumor xenografted mice model was used for evaluating FOXM1-PROTAC therapeutic response in vivo. Finally, we detected the expression of GLUT1 and PD-L1 after FOXM1-PROTAC degraded FOXM1 by using Western Blotting and hippocampal detectors and dual immunofluorescence.

**Results:**

We found that the novel FOXM1-PROTAC efficiently entered cells and induced degradation of FOXM1 protein, which strongly inhibits viability as well as migration and invasion in various cancer cell lines, and suppressed tumor growth in HepG2 and MDA-MB-231 cells xenograft mouse models, without detected toxicity in normal tissues. Meanwhile, FOXM1-PROTAC decreased the cancer cells glucose metabolism via downregulating the protein expression levels of glucose transporter GLUT1 and the immune checkpoint PD-L1, which suggests involvement of FOXM1 in cancer cell metabolism and immune regulation.

**Conclusions:**

Our results indicate that biologically targeted degradation of FOXM1 is an attractive therapeutic strategy, and antagonist peptide-containing FOXM1-PROTACs as both degrader and inhibitor of FOXM1 could be developed as a safe and promising drug for FOXM1-overexpressed cancer therapy.

**Supplementary Information:**

The online version contains supplementary material available at 10.1186/s13046-022-02483-2.

## Background

The successful invention of Proteolysis-targeting chimeras (PROTACs) has brought targeted cancer therapy into a new stage. Compared with small molecule chemotherapy drugs, monoclonal antibodies and siRNA [1, 2], PROTACs can solve the problem of drug resistance in cancer treatment to a certain extent, while having good tissue and intracellular permeability like small molecules [3, 4]. Also, PROTACs contains ligands that can specifically bind to the target protein, resulting in better targeting properties against pathogenic proteins [5, 6], especially for the degradation of enzymes [7, 8]. Moreover, new targeted protein degradation technologies such opto-PROTACs [9], peptide-based PROTACs (p-PROTAC) [10], and antibody-based PROTACs [11] are also emerging, providing more accurate and controllable treatment schemes for the treatment of cancer and other diseases. The p-PROTAC possess high specificity and low toxicity when compared with small molecule PROTAC, and can avoid the limitations of shallow binding pockets through large interacting surfaces, resulting in improving the degradation efficiency of E3 ubiquitin ligase [12].

FOXM1, one of the essential transcription factors in the forked head protein family, is ubiquitous in proliferating cells. It has been found to be highly expressed in various cancers such as liver cancer [13], lung cancer [14], breast cancer [15], and so on, and is closely related to the poor prognosis of patients [16, 17]. As a typical proliferation-related transcription factor, FOXM1 can be phosphorylated and activated by cell cycle complexes CDK4/6, promoting cell cycle transition and tissue regeneration by regulating the expression of related genes [18, 19]. It can also activate Wnt/beta-catenin [20], Raf/MEK/ERK [21], and other signal pathways to promote cell growth, metastasis, and EMT. Subsequently, it was found that FOXM1 overexpression in hepatoma cells can promote the cell cycle transition from S phase to G2/M phase and accelerate the cell cycle by regulating cell cycle genes such as, CDC25B [22] and CyclinB1 [23]. The high metabolic level of cancer cells also suggests that the expression level of GLUT1 on the cell membrane of cancer cells is higher than that of normal cells, which results in an increase in cancer cell proliferation and is consistent with the poor prognosis of cancer patients [24, 25]. Additionally, clinical data analysis shows that the expression of PD-L1 is highly correlated with the expression of FOXM1 and GLUT1 [26, 27]. However, currently there is a lack of experimental data showing a direct link between the regulation of cell proliferation by FOXM1 and the immune suppression caused by PD-L1 expression.

Here, we develop a peptide-based PROTAC, which achieves the purpose of degrading FOXM1 by facilitating the recruitment of E3 ubiquitin to FOXM1. Phage display technology was used to screen peptides in high-throughput libraries, with the peptides are effectively transported to cells by adding cell-penetrating peptide sequences. Finally, the efficient peptide is linked with the protein degradation ligand pomalidomide through appropriate PEG, forming a FOXM1-targeted protein targeting chimera named FOXM1-PROTAC. The two segments of FOXM1-PROTAC, in the presence of FOXM1 and E3 ubiquitin ligase, assist the ubiquitination and degradation the FOXM1 protein, thereby inhibiting the proliferation of liver cancer cells, further decreasing the expression of GLUT1 and PD-L1, and reducing glucose metabolism. In vivo studies confirmed that the molecule has no apparent toxicity while successfully suppressing tumors. This work suggests that FOXM1-PROTAC may become a promising cancer treatment drug.

## Methods

### Screening of FOXM1 targeting peptides

150 μl of 100 μg/ml FOXM1 full-length recombinant protein (Abnova) dissolved in NaHCO_3_ (0.1 M, pH 8.6) was added at the 96-well microplate, The well plate was then rotated until the surface is completely entirely wetted and then incubated overnight in a humidified container at 4 °C with agitation. The next day, an ER2738 individual clone was put into 30 ml lysogeny broth (LB) medium and cultured under vigorous shaking at 37 °C. Simultaneously, we discarded the coating liquid in the microtiter plate, shook off the remaining liquid, filled the plate with the blocking solution immediately, and incubated at 4 °C for 1 h. Afterward, the blocking solution was discarded, and TBST (50 mM pH 7.5 Tris-HCl, 150 mM NaCl, 0.1% Tween-20) buffer was used to wash the plate six times. 10 μl of (2 × 10^11^ PFU) Ph.D.™ phage library (BioLabs) was added to 100 μl TBST, transferred to a microplate, and shaken at room temperature for 1 h. The nonbinding phages were discarded, and the microplate was washed ten times with TBST buffer. After removing residual water from the plates, 100 μl Glycine-HCl (0.2 M, pH 2.2) was added and the plate was rocked gently for 15 minutes to obtain the binding phages. 15 μl Tris-HCl (1 M, pH 9.1) was used to neutralize the eluate. 2 μl of the eluate was taken out, and the remaining eluate was put into 20 ml of ER2738 bacterial solution (approximate OD_600_ value of 0.5), shaken vigorously at 37 °C for 5 h. The culture was transferred to a clean centrifuge tube and centrifuged at 4 °C, 10,000 rpm for 10 minutes. The supernatant was transferred to a fresh centrifuge tube where PEG/NaCl (20% PEG-8000, 2.5 M NaCl) solution was added at approximately 1/6th the volume of the supernatant and was then allowed to precipitate overnight at 4 °C. The precipitate was then centrifuged at 4 °C, 10,000 rpm for 15 min. The pellet was resuspended in 1 ml TBST and centrifuged at 4 °C, 10,000 rpm for 5 minutes. Another 1/6th volume of PEG/NaCl was added to the pellet and incubated on ice for 1 h, then centrifuged at 4 °C, 10,000 rpm for 10 minutes. The precipitate was then resuspended in 200 μl TBS as the amplification eluate, and could be used for the second round of screening.

Meanwhile, the spare 2 μl eluate was diluted 10^1^–10^4^ fold separately, and 10 μl eluate of different dilutions were added to 200 μl of ER2738 bacterial solution (approximate OD_600_ value of 0.5), mixed, and incubated at room temperature for 5 minutes. The infected cells were added to 3 ml top agar preheated to 45 °C, mixed, and immediately poured into the LB/IPTG/Xgal plate preheated to 37 °C. The plate was incubated overnight at 37 °C after cooling. The plates with approximately 100 plaques had the plaque counted and then multiplied by the dilution factor to obtain the phage titer in plaque forming unit (PFU) titer of phage per 10 μl. According to this value, the addition amount of corresponding 2 × 10^11^ PFU was calculated, and an additional two or three rounds of screening were carried out according to the above steps. In the second and third rounds of screening, 15 clones were selected from the plates with less than 100 plaques. After amplification according to the above amplification steps, phage sheath DNA was extracted with M13 phage DNA Extraction Kit (Omega Bio-tek) and sequenced.

### Synthesis of peptides and proteolysis-targeting chimeras

The three peptides obtained from the phage library were further conjugated by solid-phase synthesis (Apeptide, Shanghai). Specifically, the final three peptides were conjugated with FITC and TAT (GRKKRRQRRRPPQQ) cell-penetrating peptides at the N-terminus and C-terminus. The synthesized peptides were purified to a purity greater than 95% using HPLC. Lastly, MS detection was performed. When used the peptide needs to be resuspended in sterile double-distilled water, and then stored at − 20 °C. The pomalidomide-PEG_2_-COOH was synthesized chemically (Biochempartner, Shanghai), purified to a purity greater than 98% using HPLC, and subjected to MS detection. FOXM1-PROTAC was also synthesized via chemical methods (Apeptide, Shanghai), purified to a purity greater than 95% using HPLC, and tested by MS. When ready for use, it was resuspended in sterile double-distilled water and stored at − 20 °C.

### Cell lines and cell culture

HepG2, A549, MDA-MB-231 and HCT116 cells were obtained from ATCC (Manassas, USA). All cells were grown in Dulbecco’s Modified Eagle Media (Gibco) medium and supplemented with 10% fetal bovine serum (Gibco). Unless otherwise specified, all cell cultures were grown at 5% CO_2_, 37 °C.

### Confocal fluorescence microscopy analysis of cell uptake and localization of FIP-1

HepG2 cells were seeded in a 12-well plate at 5 × 10^4^ cells/well with cell climbing films (NEST) and allowed to culture overnight. The next day, the medium was discarded and the cell climbing films were washed three times with cold PSB. The FITC-labeled peptide was diluted with water to make a 1000 μM mother solution according to the molecular weight, and with further dilution a final peptide concentration of 10 μM was added to the plates, and incubated at 37 °C for 6 h. Subsequently, the culture medium was discarded, rinsed three times with cold PBS, and fixed with 4% paraformaldehyde for 10 minutes at room temperature. The cells were first washed three times with 0.01% PBST (PBS, 0.01% Triton-100), and then 0.2% PBST (PBS, 0.01% Triton-100) was added and the cells were incubated for 10 minutes at room temperature. Then the cells were blocked with 2% BSA (PBS, 2% BSA) for 30 minutes at room temperature. After discarding the blocking solution, the cells were washed three times with 0.01% PBST. The FOXM1 antibody (CST) was then added according to the appropriate proportion and the cells were incubated at room temperature for 2 hours. Excess antibody was discarded, and the cells were washed three times with 0.01% PBST. Fluor 555-labeled fluorescent secondary antibody (CST) was added and the cells were incubated for 1 h at room temperature. Excess secondary antibody was aspirated, and then the cells were washed thrice with PBS, 3 μM DAPI (Invitrogen) was then added, and the cells were incubated afterwards for 10 minutes at room temperature. Cell climbing films were taken out, and was fixed with a fixative containing anti-fluorescence quencher (Corning). The final images were taken under a two-photon fluorescence microscope (Nikon).

### Cell viability

HepG2 cells were seeded at 5 × 10^3^ per well in 96-well plates and cultured overnight. The following day, the medium was then replaced, and different concentrations of peptides or FOXM1-PROTAC were added. The culture plate was incubated in the incubator for 48 h, and then 10 μl of CCK-8 solution (Abcam) was added to each well. After incubating for 1–4 h, and the absorbance at 450 nm was measured with a microplate reader. The cell viability was calculated according to the following formula, and the inhibition curve also was drawn. Cell viability (%) = [A (dosing)-A (blank)]/[A(0dosing)-A (blank)] × 100, A (dosing): the OD value of the wells with cells, CCK-8 solution, and drugs. A (0 dosing): the OD value of the wells with cells and CCK-8 solution but no drug solution, A (blank): the OD of the wells without cells.

### Colony formation assay

HepG2 hepatocellular carcinoma cells were seeded in a 6-well cell culture plate at 100 cells/well, with a medium volume of 2 ml per well, and were cultured for 24 h, after culturing, 20 μM FOXM1-PROTAC and the appropriate amount of fresh medium were added. The peptides were introduced to the experimental groups while no drugs were added to the control group. The medium and drugs were replaced every 2 days, and the cells were cultured continuously until each cell cluster contained more than 50 cells. After fixing the cells in the experimental and control groups with methanol, the cells in both were stained with 0.1% crystal violet (Macklin). The number of clones formed in each group was then counted.

### Scratch assay

HepG2 was inoculated into a new 6-well cell culture plate with a ratio of 1:2. 2 ml of medium was added to each well. After 24 h of culture, the cells were scratched with a wall, and the floating cells and debris were washed away with PBS. The scratch distance was photographed and recorded with a microscope, FOXM1-PROTAC with a concentration of 20 μM was added to the plate and an appropriate amount of serum-free fresh medium was used as the control group. After 48 hours, the floating cells were washed off, and the scratch distance was recorded by microscope.

### Transwell

One hour before the experiment, Matrigel (Corning) was mixed with medium according to the protocol, and then added to the upper well and cultured at 37 °C, 5% CO_2_ for 1 h. After gel coagulation, HepG2 and MDA-MB-231 cells were diluted with serum-free DMEM to obtain 5 × 10^5^ cells in each well. FIP-1 and FOXM1-PROTAC with a concentration of 20 μM were added. At the same time, DMEM with 20% serum was added to the lower well, and was incubated at 37 °C, 5% CO_2_. 24 h later, wash with PBS to remove the residual medium, fix with paraformaldehyde for 20 minutes, and gently wipe the non-transferred cells on the membrane with a cotton swab. After crystal violet staining for 20 minutes, wash with PBS until the background is colorless, take photos under the microscope, and count.

### Protein extraction and Western blotting

HepG2 hepatocellular carcinoma cells were seeded at 5 × 10^5^ cells/well in a 6-well cell culture plate, and 2 ml medium was added to each well to culture overnight. Different concentrations of FIP-1 or FOXM1-PROTAC were added, and the cells were collected 24 h later; in the time group, 20 μM FIP-1 and FOXM1-PROTAC were added, and the cells were collected at set times. The collected cells were extracted with the total cell protein extraction kit (Thermo Fisher), and the total protein concentration was determined with the Pierce BCA Protein Assay Kit (Thermo Scientific). The 10% SDS-PAGE was prepared in advance with the PAGE Gel Fast Preparation Kit (Omni), and then the sample was analyzed. FOXM1, GLUT1, and PD-L1 antibodies (CST) were used to detect the corresponding proteins, and β-actin (CST) was used as an internal reference for quantitative analysis.

### Real-time PCR

After treatment with 20 μM peptide and FOXM1-PROTAC for 24 h, the total RNA of the cells was extracted with TRIzol™ Plus RNA Purification Kit I (Thermo Scientific). Immediately, total RNA was reverse transcribed using High-Capacity cDNA Reverse Transcription Kit (Thermo Scientific) in a 42 °C metal bath. The DyNAmo Color Flash SYBR Green qPCR kit (Thermo Scientific) was used to perform quantitative real-time PCR using the Bio-Rad machine, using the FOXM1 primers (5′-ATACGTGGATTGAGGACCACT-3′5’-TCCAATGTCAAGTAGCGGTTG-3′), and the experimental results were analyzed.

### Flow cytometry analysis of the cell cycle and PD-L1 expression

About 2 × 10^6^ HepG2 cells were seeded in 6 cm plates and cultured overnight. Then the medium was removed, FIP-1 or FOXM1-PROTAC at a final concentration of 20 μM were added to the fresh medium, and the cells were cultured for another 24 h. After that, the cells were processed according to the Cell Cycle and Apoptosis Analysis Kit (Beyotime) protocol, and the cell cycle was detected using the flow cytometry (Beckman). Similarly, HepG2 cells, treated with FIP-1 or FOXM1-PROTACs, were stained with PD-L1–488 (CST) antibody and analyzed by flow cytometry.

### Lactate analysis

1 × 10^6^ HepG2 cells were seeded in 3.5 cm plates and cultured overnight. Then the medium was removed and the plates were washed twice with cold PBS, replaced with fresh medium and FIP-1 or FOXM1-PROTAC at a final concentration of 20 μM were added. After culturing for 24 h, the cells were collected, 200 μl of Lactate Assay Buffer was added, and then the cells were well shaken and mixed. Then the collected cells were centrifuged at 12000 g for 5 minutes at 4 °C, and the supernatant was drawn for lactate analysis. The detection reagents were configured according to the CheKine™ Lactate Assay Kit (Abbkine). The OD value at 450 nm was measured at the start and 30 minutes later, and the lactate metabolism level was analyzed.

### 2-NBDG uptake assays

2 × 10^6^ HepG2 cells were inoculated in 6 cm plate. After culturing overnight, the medium was replaced with fresh medium, and FIP-1 or FOXM1-PROTAC at a final concentration of 20 μM were added. After 24 h of treatment, the medium was discarded and washed twice with cold PBS. A sugar-free and serum-free medium was used, and 2-NBDG with a final concentration of 300 μM was added to the plate. After 30 minutes of treatment, the medium was aspirated and washed with cold PBS until there was no residual 2-NBDG. The uptake of glucose was recorded by microscope (Nikon). At the same time, the cells were collected and resuspended in 200 μl of pre-cooled PBS and then the propidium iodide (PI) concentration was adjusted to 1 μg/ml, and kept at 4 °C for 30 minutes. Flow cytometry analysis was then performed.

### Metabolic assays

The 96-well plate was seeded with 2 × 10^4^ cells per well, an appropriate amount of medium was added, and the cells were allowed to culture overnight. The next day, the medium was replaced, and the experimental groups were treated with 20 μM FIP-1 or FOXM1-PROTAC. The control group was instead treated with the sterile water and cultured. After culturing for 24 h, the medium was aspirated and the Glycolysis Assay (Extracellular acidification) kit (Abcam) was used according to the set procedures. The prepared plate was then put into the fluorescent plate reader preset to a temperature of 37 °C. The glycolysis test signal was measured every 1.5 minutes for at least 2 h, using excitation and emission wavelengths of Ex/Em = 380/615 nm.

### Tumorigenesis assays in nude mice

We purchased 21 female BALB/c nude mice (female, 6-week old) from Guangdong Medical Laboratory Animal Center. HepG2, a human hepatocellular carcinoma cell line, was used in a subcutaneous tumor model. Each nude mouse was injected subcutaneously with 5 × 10^6^ HepG2 cells for tumor growth (approximately one week). When the tumor volume exceeded 50 mm^3^, mice were randomly assigned, seven per group, into the control group, FIP-1 group, and FOXM1-PROTAC group. The FIP-1 group and FOXM1-PROTAC group were injected with 20 mg/kg FIP-1 and FOXM1-PROTAC, respectively, through the caudal vein once a day for 14 days. The tumor volumes and nude mice body weights were measured every 2 days. The tumor volumes and nude mice weight continued to be monitored the week after halting treatment. Then, after collecting blood from the mice via retro-orbit, the tumor and several organs were removed and fixed in 4% paraformaldehyde. Tissue sectioning, immunohistochemistry, and immunofluorescence experiments were outsourced to a company (Servicebio). The blood was stored at 4 °C overnight and then centrifuged at 4 °C, 8000 rpm for 5 minutes. The supernatant was collected, and a kit was used to detect the liver index.

### In vivo biodistribution

Ten female BALB/c subcutaneous tumor mice were randomly divided into two groups with five animals in each group. Then 20 mg/kg FIP-1 and FOXM1-PROTACs were injected through the caudal vein. Peripheral blood was taken at 6 h, 12 h, 24 h and 48 h respectively, and the tumors and organs were collected after 48 h. They were homogenized and lysed with RIPA buffer added with protease inhibitor cocktail. The distribution of FOXM1-PROTACs in blood and organs was detected with PEG- ELISA kit (Life Diagnostics).

### Statistical analysis

Microsoft Excel program or Graphpad Prism 7 was used to calculate the mean ± standard deviation of the sample. For the analysis method, we used unpaired two-tailed student’s t-tests to analyze the differences between the two groups. One-way ANOVA followed by Bonferroni’s multiple comparison tests was used for multiple comparison test. Statistical significance was defined as *∗∗∗p* < 0.001; *∗∗p* < 0.01; *∗p* < 0.05; n.s. = not significant.

## Results

### High expression of FOXM1 is associated with poor cancer prognosis and with the expression of GLUT1 and PD-L1

**Fig. 1:**
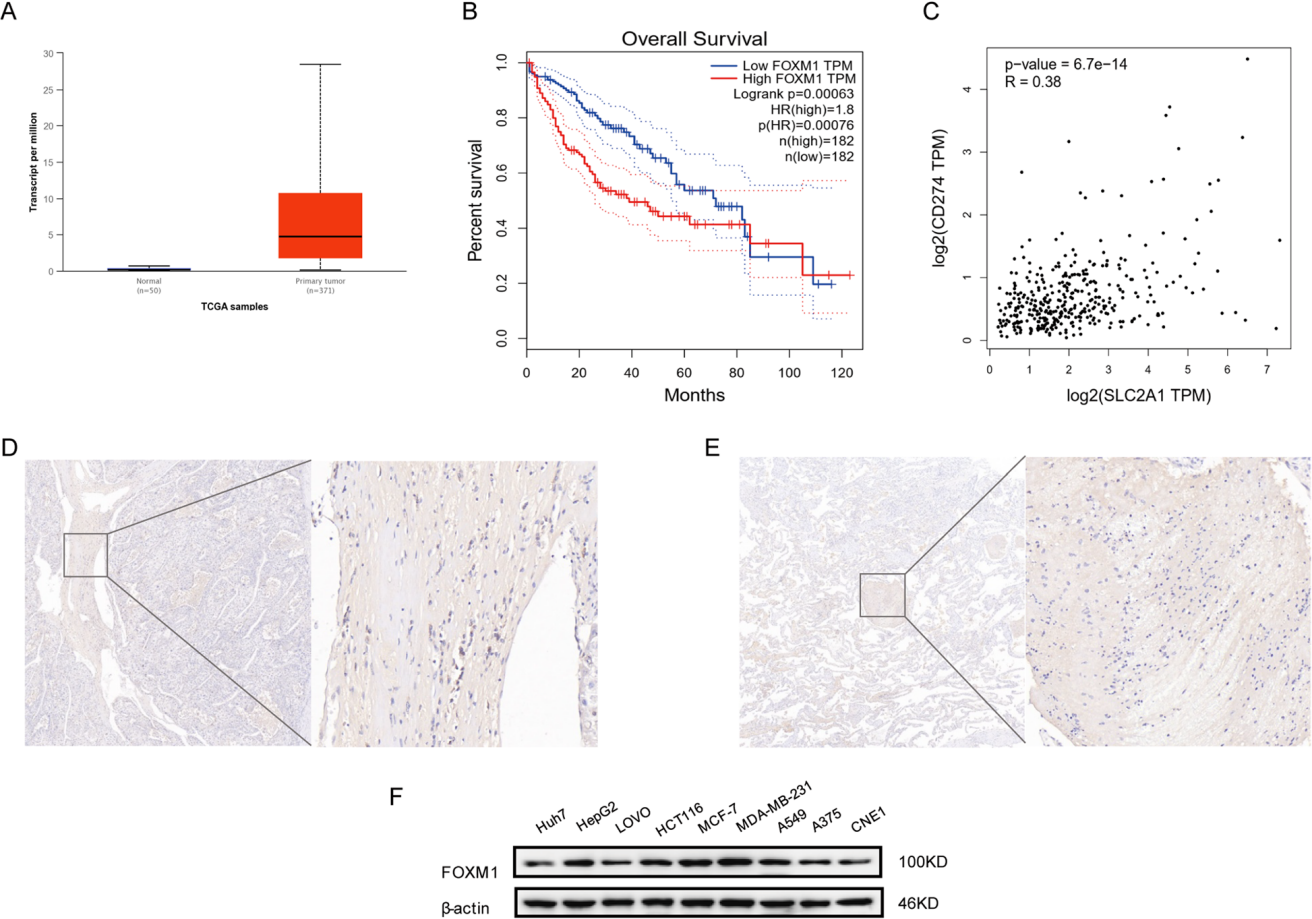
High expression of FOXM1 in cancers is closely correlated with poor prognosis, and with the expression of GLUT1 and PD-L1. **A** Expression of FOXM1 in hepatocellular carcinoma samples. **B** Relationship between FOXM1 expression and prognosis in hepatocellular carcinoma. **C** Correlation between GLUT1 and PD-L1 expression in hepatocellular carcinoma. **D** Expression of GLUT1 in hepatocellular carcinoma samples. **E** Expression of PD-L1 in hepatocellular carcinoma samples. **F** Detection of FOXM1 protein expression in different cancer cell lines

In order to clarify the relationship between FOXM1 and cancer in patients, we took liver hepatocellular carcinoma as an example to validate the differences in expression between normal tissues and tumors. It was found that the expression of FOXM1 in tumor cells was several times higher than that in normal tissue cells (Fig. 1A). Based on this, we analyzed the impact of differential expression of FOXM1 on the prognosis of cancer patients [16, 17]. The results showed that the high expression of FOXM1 was significantly correlated with the poor prognosis of cancer patients (Fig. 1B). Additionally, we researched the correlation between FOXM1 downstream gene GLUT1 and immune checkpoint PD-L1 [26–28]. The data presented a notable correlation between PD-L1 and GLUT1 (Fig. 1C). Then, we further confirmed the high expression of FOXM1 and PD-L1 by immunohistochemical staining of tumor tissues and paracancerous tissues in five liver cancer patients. This high expression may be related to the occurrence and development of cancer and the prognosis of cancer patients (Fig. 1D, E). Subsequently, we investigated the expression of FOXM1 in different cancer cell lines to determine the universality and importance of FOXM1 expression. Western Blotting results revealed that FOXM1 is also highly expressed in cell lines of other cancers besides hepatocellular carcinoma (Fig. 1F), which could be effective models for follow-up studies and related research.

### Peptides that specifically bind to FOXM1 inhibit cancer cells growths

**Fig. 2:**
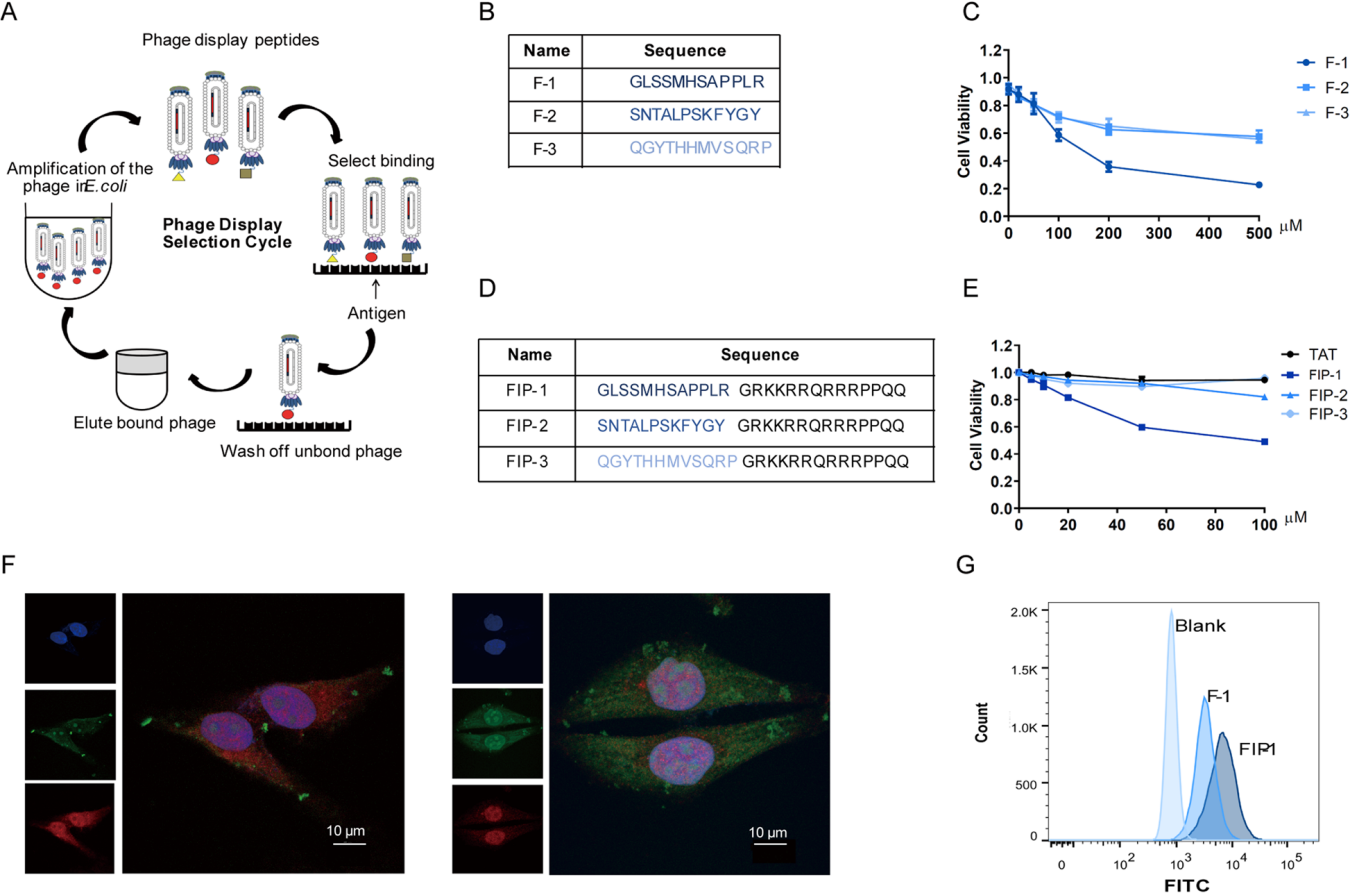
FIP-1 binds to FOXM1 and inhibits viability of cancer cells. **A** Screening and identification of FOXM1 targeted peptides using an in vitro phage display. **B** Name and sequence of FOXM1 targeted peptides. **C** Effect of three rounds of screening peptides was verified by CCK8 in HepG2 cells. **D** Peptides were renamed after labeling the TAT sequence at the C-terminal. **E** Viability of cells, treated with three screened peptides for 24 h, were performed using CCK-8, and the results showed that FIP-1 inhibited the growth of HepG2 cells. **F** Localization of FOXM1 (red) and FITC-labeled FIP-1 (green) was imaged with fluorescent Con-focal microscopy. Cell nuclei was stained by DAPI (blue). Lift: F-1, Right: FIP-1. **G** The level of cellular uptake was quantified with flow cytometry

To develop peptide-based proteolysis-targeting chimeras [29, 30], we screened peptides using peptide phage display libraries and FOXM1b full-length protein, which is the shortest-length effective protein in the FOXM1 family, according to the phage screening process (Fig. 2A). After three rounds of screening, we analyzed the results of the second and third rounds of screening (Fig. S1A, B, Supporting Information). By aligning the sequences, we obtained three peptides targeting FOXM1 (Fig. 2B). As solubility plays a corresponding role in subsequent experiments, we then analyzed the hydrophobicity of the three peptides, with the results showing that the three peptides had good water solubility (Fig. S1C, Supporting Information). Cell viability experiments were carried out to verify the inhibitory effect of the peptides on hepatoma cells. We found that F-1 had better inhibitory effect than other peptides (Fig. 2C). Although some peptides are capable of entering cells without a carrier, most peptides have limited permeability across the cell membrane. To ensure that our peptides can successfully cross the cell membrane and reach a high intracellular concentration in the cell, we selected the cell membrane penetrating peptide TAT [31, 32] as the carrier to aid our small molecule PROTAC into the cell. Moreover, we found that the position of TAT did not affect its membrane- penetrating property [33]. Therefore, we placed the TAT sequence at the N-terminal of the peptides, resulting in three synthesized peptides with membrane penetrating properties (Fig. 2D). Next, we focused on the binding ability of the three peptides with FOXM1.The molecular docking of FOXM1b protein with FIP-1 also confirmed that they could overlap, and the binding position may be in the transcriptional activation domain of FOXM1b protein (Fig. S1D, Supporting Information). Finally, we sought to determine whether the addition of different concentrations of FIP-1 to FOXM1 could inhibit the activity of FOXM1 and affect its function. It was found that FIP-1 has a significant inhibitory effect on cells (Fig. 2E). Fluorescence confocal experiments demonstrated that N-terminal FITC labeled FIP-1 could not only enter cells successfully, but also bind to FOXM1 effectively (Fig. 2F). Additionally, we quantified and statistically analyzed the fluorescence intensity of peptides by flow cytometry, which verified peptides with TAT were more easily absorbed (Fig. 2G). Together these data indicated that FIP-1 peptide binds to FOXM1 and significantly inhibits the viability of cancer cells as an antagonist of FOXM1.

### FOXM1-PROTAC induces FOXM1 protein degradation and decreases viability of cancer cells in a dose-dependent manner

**Fig. 3:**
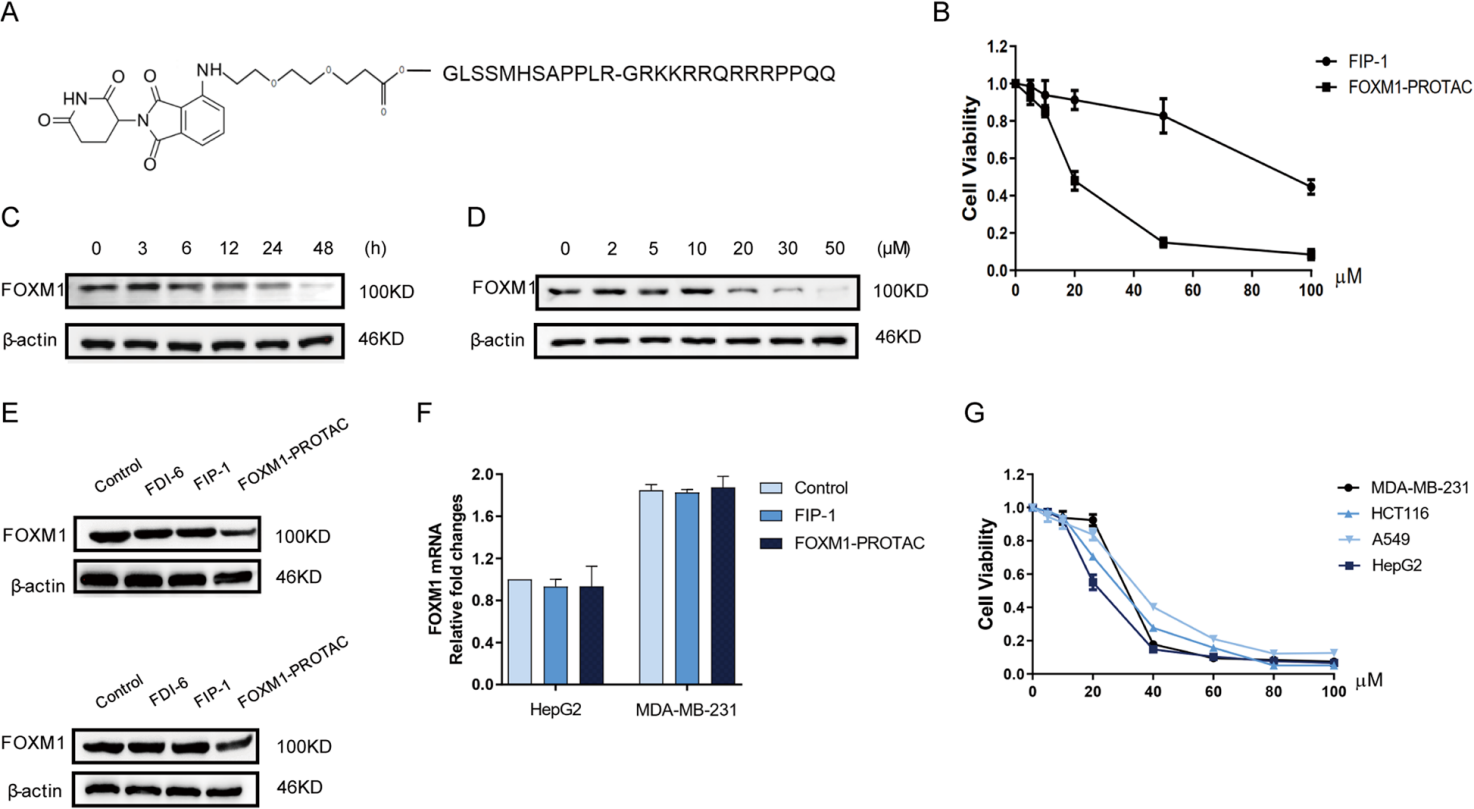
FOXM1-PROTAC induces FOXM1 protein degradation. **A** Structure of FOXM1-PROTAC. **B** Viability of cells, treated with FIP-1 and FOXM1-PROTAC for 24 h, were performed using CCK-8, and the results showed that FOXM1-PROTAC has a better inhibitory effect on cells. **C**, Western blotting examination for FOXM1 of HepG2 cells treated with 20 μM FOXM1-PROTAC for different time (0, 3, 6, 12, 24, 48 h). **D** Western blotting test for FOXM1 of HepG2 cells treated for 24 h with increasing concentrations of FOXM1-PROTAC (0, 2, 5, 10, 20, 30, 50 μM). **E** Expression of FOXM1 in HepG2 and MDA-MB-231 cells treated with 20 μM FDI-6, FIP-1 and FOXM1-PROTAC for 24 h. **F** Detection of FOXM1 mRNA by Real-time PCR in HepG2 and MDA-MB-231 cells treated with 20 μM FOXM1-PROTAC for 24 h. **G** HepG2, A549, MDA-MB-231, HCT116 cells viability, treated with 20 μM FOXM1-PROTAC for 24 h respectively, were performed using CCK-8

Previous work had revealed that the length of the E3 ubiquitin ligase [34, 35] recruitment element and linker would affect the degradation [36]. We reasoned that pomalidomide [37] could act as the recruitment element of E3 ubiquitin ligase to achieve degradation, and so Pomalidomide-PEG_2_-COOH with PEG_2_ as a linker was synthesized. Then Pomalidomide-PEG_2_-COOH was chemically linked with the peptide containing TAT sequence, which was the FOXM1 targeting PROTAC named FOXM1-PROTAC (Fig. 3A and Fig. S2A, Supporting Information). We next investigated whether the peptide linked with pomalidomide could exert inhibitory function. Cell viability experiments were conducted to detect the inhibitory effect of FOXM1-PROTAC on Hepatoma cell HepG2. The results show that FOXM1-PROTAC had a more potent inhibitory effect on cancer cell viability than peptide FIP-1 (Fig. 3B), which is expected as FIP-1 inhibits FOXM1 only by binding to FOXM1, whereas FOXM1-PROTAC eliminates the majority of FOXM1 and can bind to the remaining nondegraded FOXM1. Naturally, we explored the difference in FOXM1 degradation in HepG2 cells induced by FOXM1-PROTAC versus FIP-1. FOXM1-PROTAC adjusted to a final concentration of 20 μM was added to HepG2 cells, with the cells being collected at different times to extract total protein for western blotting. Compared to the FIP-1 peptide group, the FOXM1-PROTAC group saw a gradual decrease in total FOXM1 protein (Fig. S2B, Supporting Information), and the degradation efficiency had exceeded 50% by 24 h (Fig. 3C). Furthermore, we investigated the effects of different concentrations of FOXM1-PROTAC on FOXM1 protein degradation. We added different concentrations of FOXM1-PROTAC to HepG2 cells, collected and detected the total cell protein after 24 h. Results suggested that varying concentrations of FOXM1-PROTAC caused different levels of degradation of the FOXM1 protein, with increased degradation at higher concentrations of FOXM1-PROTAC (Fig. 3D and Fig. S2C, Supporting Information). An experiment was conducted to confirm that FOXM1-PROTAC was the cause of the decrease in FOXM1 protein. We selected FOXM1 inhibitor FDI-6 [38] as the negative control and compared FOXM1 protein levels in HepG2 and MDA-MB-231 cell lines following treatment. Our synthesized PROTAC was confirmed to cause the decrease in FOXM1 protein levels (Fig. 3E). In order to exclude the possibility of varied gene expression levels as the cause of differing FOXM1 protein levels, RNA was extracted from HepG2 and MDA-MB-231 cell lines 24 h after treatment with 20 μM FOXM1-PROTAC. The FOXM1 RNA was detected using qPCR and the results indicated that the decreased amount of FOXM1 protein occurred due to degradation caused by FOXM1-PROTAC, not from a reduction of RNA caused by gene expression level (Fig. 3F). Additionally, we verified that FOXM1-PROTAC had the same inhibitory effect on different cancer cell lines (Fig. 3G). In this part of the experiment, we successfully developed a FOXM1-specific targeting PROTAC, which reduced FOXM1 protein degradation and inhibited cell viability.

### FOXM1-PROTAC inhibits proliferation, migration and invasion of cancer cells

**Fig. 4:**
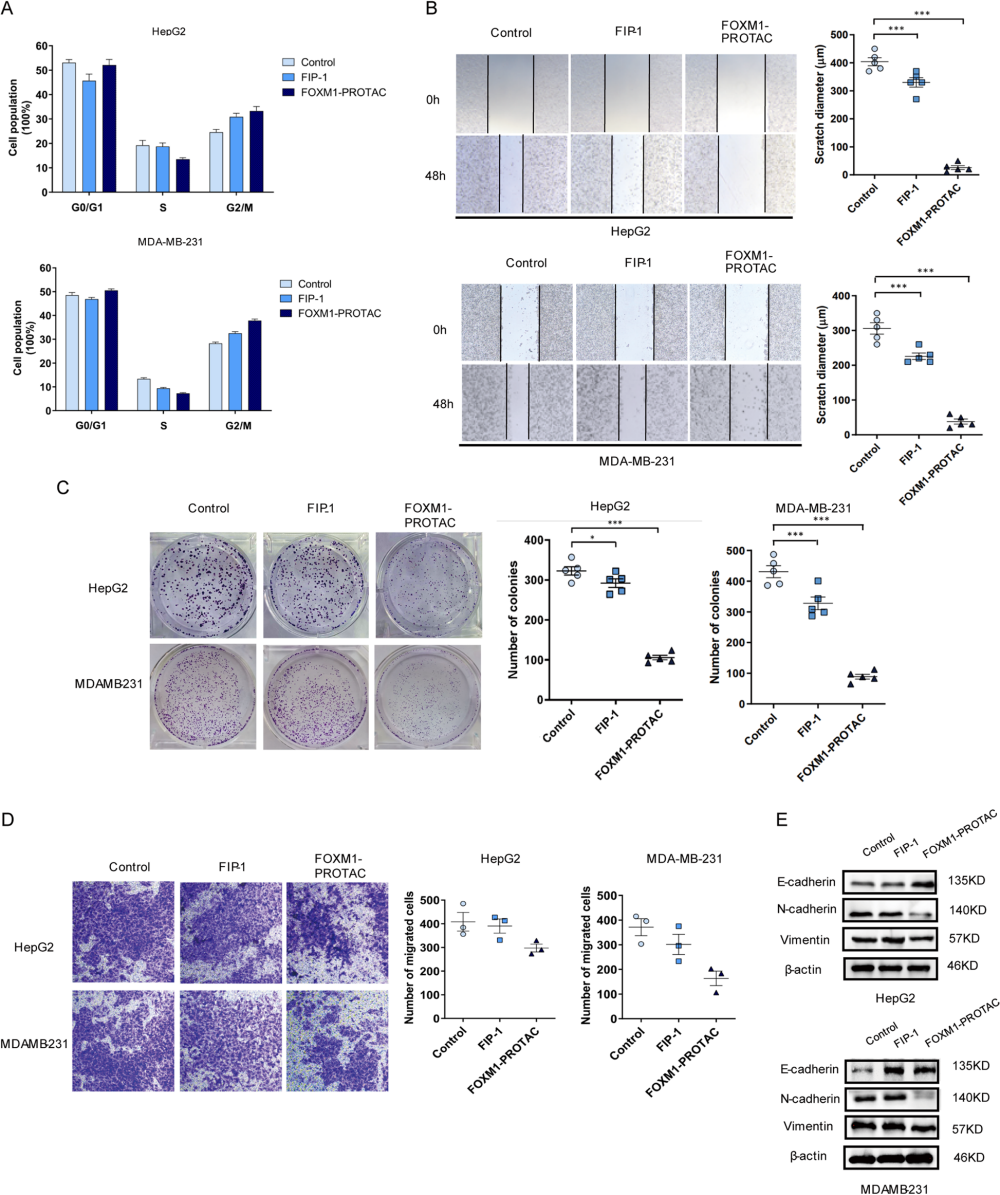
FOXM1-PROTAC inhibits proliferation, migration and invasion of cancer cells. **A** Analysis for DNA content of HepG2 and MDA-MB-231 cells on a Flow cytometer, treated with FIP-1 and FOXM1-PROTAC for 24 h and stained with propidium iodide. **B** Scratch assay of HepG2 and MDA-MB-231 cells, which were treated with FIP-1 and FOXM1-PROTAC for 24 h. **C** Colony formation results and analysis of HepG2 and MDA-MB-231 cells, which were treated with FIP-1 and FOXM1-PROTAC for 24 h and cultured for 14 days. **D** Transwell experiment and Statistical histogram, which reflects different invasion ability of HepG2 and MDA-MB-231 cells treated with FIP-1 and FOXM1-PROTAC for 24 h. **E** Examination of E-cadherin, N-cadherin and Vimentin level of HepG2 and MDA-MB-231 cells, treated with FIP-1 and FOXM1-PROTAC for 48 h

Based on the prior experimental results, we focus on the effect of FOXM1-PROTAC on the growth of Hepatoma cells and Breast cancer cells. As a vital proliferation-related gene [39], FOXM1 plays a crucial role in regulating the cell cycle. To explore its effect on the cell cycle, FOXM1-PROTAC, at a concentration of 20 μM, was used to treat HepG2 and MDA-MB-231 cells. The cells were collected 24 h later, and their cell cycle was detected using flow cytometry. As shown in Fig. 4A and Fig. S3A, Supporting Information, following treatment, the cell population in the S phase decreased and the number of cells in the G2/M phase increased relatively, indicating that FOXM1-PROTAC can suppress FOXM1-regulated the cell cycle transition from S phase to G2/M phase. This phenomenon is consistent with the expression of cell proliferation-related markers, such as PCNA [40], Ki-67, and CDC-25B (Fig. S3B, Supporting Information). The ability of FOXM1-PROTAC in suppressing migration was confirmed through a scratch experiment. Compared with the control group, the scratch distance of the FOXM1-PROTAC-treated group was wider, exhibiting that the migration ability of cells was relatively weaker (Fig. 4B). In addition, we tested whether the synthesized FOXM1-PROTAC had an effect on the tumorigenicity of cancer cells. A colony formation experiment was conducted and after the set 14 days period, the number of colonies were significantly decreased after treating with FOXM1-PROTAC in HepG2 and MDA-MB-231 cells (Fig. 4C). We then confirmed that 20 μM FOXM1-PROTAC could effectively inhibit cell invasion using a transwell assay experiment. The statistical results showed that the number of invasive cells in the FOXM1-PROTAC-treated group decreased significantly (Fig. 4D). For migration-related gene expression, the FOXM1-PROTAC treatment group saw an increased expression level of E-cadherin, and a decreased expression levels of N-cadherin and vimentin (Fig. 4E).

### FOXM1-PROTAC suppresses tumor growth in vivo with little toxicity to normal tissues

**Fig. 5:**
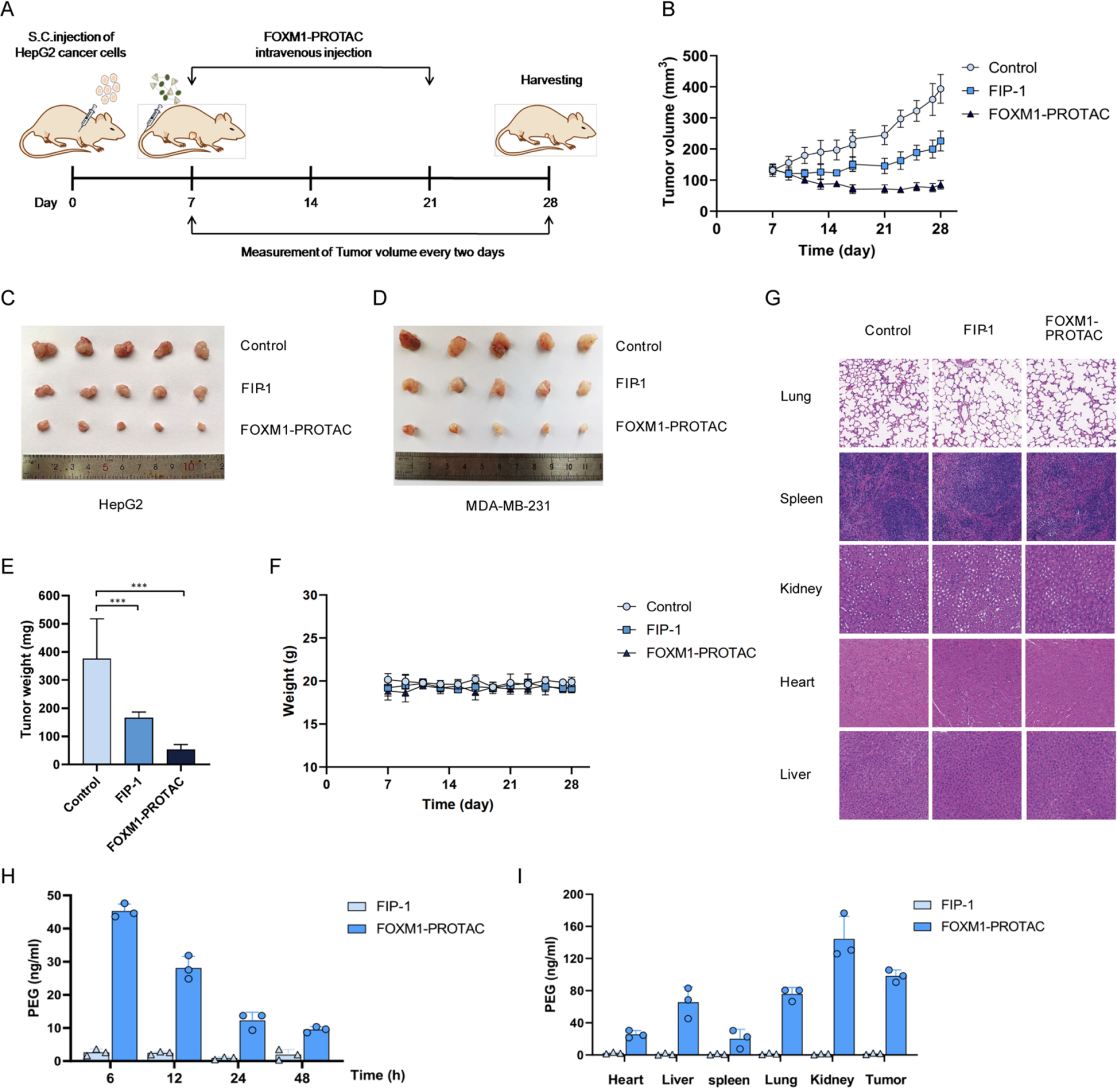
FOXM1-PROTAC suppresses tumor growth in vivo. **A** The protocol of FOXM1-PROTAC treatment in vivo. BALB/c nude mice (*n* = 21) were subcutaneously (S.C.) injected with HepG2 and MDA-MB-231 cells (10^6^ cells/mouse) into the right axilla. When the tumor volumes reached 50–100 mm^3^, the mice were randomly divided into three group. FIP-1 or FOXM1-PROTAC (20 mg/kg) was injected directly into tumors (caudal vein injection) once a day for 14 days. The size of engrafted tumors was measured at the interval of two days and tumor samples were collected at Day 28 post the injection of cancer cells. **B** Changes of tumor volume with administration days in mice injecting with HepG2 cells. **C** Hepatoma tumor picture after 28 days. **D** Breast tumor picture after 28 days. **E** Statistical diagram of tumor volume of mice injected with HepG2 cells in experimental group and control group. **F** Changes of body weight of mice injected with HepG2 cells. **G**, Immunohistochemistry of Heart, Liver, Spleen, Lung and Kidney Treated by FIP-1 and FOXM1-PROTAC for 14 Days (20 mg/kg). H, Serum ELISA of xenografted mice treated by FIP-1 and FOXM1-PROTACs (20 mg/Kg). I, ELISA of Heart, Liver, Spleen, Lung and Kidney treated by FIP-1 and FOXM1-PROTACs (20 mg/kg)

We established the transplanted tumor model of 21 nude mice by subcutaneous injection of HepG2 and MDA-MB-231 cells. One experimental group was injected with 20 mg/kg FOXM1-PROTAC, the other was injected with 20 mg/kg FIP-1, and the control group was injected with the same volume of sterile water for 14 days. During this period, the weight of the nude mice and the volume of the tumor were measured every 2 days, with the measurements continuing for 7 days after injections were stopped to observe the recovery (Fig. 5A). On the 28th day, blood was taken from the orbit of each nude mice, and they were then humane lyeuthanized. The heart, liver, spleen, lung, kidney, and tumor were collected. The statistical analysis of tumor size data revealed that the tumor volume of the FOXM1-PROTAC experimental group was much smaller than that of the control group, indicating that FOXM1-PROTAC had a significant inhibitory effect on tumor growth (Fig. 5B and Fig. S4A, Supporting Information). The tumors from the three treatment groups were weighed. We found that tumors from both cancer cells lines treated with FOXM1-PROTAC were significantly smaller than those in the other two groups (Fig. 5C, D, E). We also analyzed the changes in body weight of the mice throughout treatment protocol and found that the weight of the mice remained steady in both the experimental groups and the control group, indicating that the drug toxicity was low and had little effect on their body weight (Fig. 5F and Fig. S4B, Supporting Information). To further investigate toxicity, we performed immunohistochemical staining on various tissues and found that FOXM1-PROTAC had almost no toxicity, which was consistent with our previous conclusion based on the stable body weights of the nude mice (Fig. 5G and Fig. S4C, Supporting Information). We tested the liver indexes with the extracted serum and determined that FOXM1-PROTACs had almost no side effects (Fig. S4D, E, Supporting Information). Finally, the biodistribution of injected FOXM1-PROTACs was implemented by ELISA (Fig. 5H, I). Between 0 h and 12 h, we observed a moderate decrease in serum FOXM1-PROTACs. Twelve hours after injection, there was a dramatical reduction, and tissue distribution was observed to be liver, tumor, lung, kidney, heart in descending order.

### FOXM1-PROTAC inhibits the glucose metabolism through downregulating the expression of GLUT1 and PD-L1

**Fig. 6:**
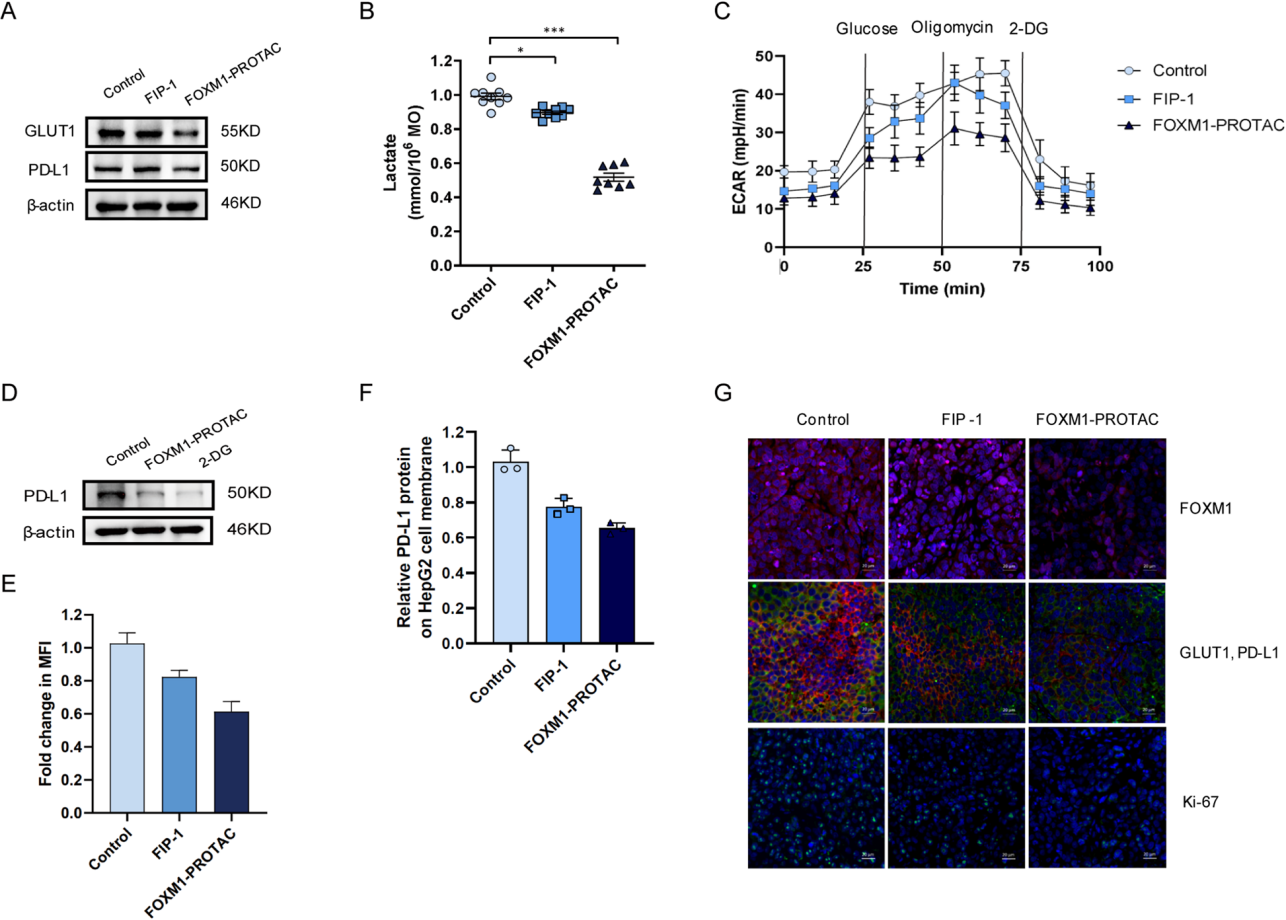
FOXM1-PROTAC inhibits the expression of GLUT1 and PD-L1. **A** Detection of PD-L1, GLUT1 in HepG2 cells treated with FIP-1 and FOXM1-PROTAC for 24 h. **B** Decreasing lactate level in HepG2 cells, treated with FIP-1 and FOXM1-PROTAC for 24 h, was showed by Microplate Reader. **C**, Seahorse extracellular flux analyzer (SEFA) measurement of ECAR metabolic profile in HepG2 cells treated with FIP-1 and FOXM1-PROTAC for 24 h. **D** Expression of PD-L1 in HepG2 cells after treating with 2-DG for 24 h. **E** HepG2 cells treated with FIP-1 and FOXM1-PROTAC for 24 h uptake analysis of 2-NBDG (200 μM) on a Flow cytometer. **F** Flow cytometer analysis of PD-L1 expression on tumor cell surface before or after FOXM1-PROTACs treatment. **G** Immunofluorescence of tumor samples, FOXM1 (red single), GLUT1 and PD-L1 (red and green merged), ki-67 (green single) and Cell nuclei was stained (blue)

Previous research reported that FOXM1 could bind to the *GLUT1* promoter [28, 41], that inhibition of FOXM1 could inhibit *GLUT1* expression, and that FOXM1 expression was highly correlated with PD-L1 expression in patients. To further explore the relationship between FOXM1, metabolism, and immunity, we investigated the expression of GLUT1, an essential transporter in the intracellular glucose transporter family, and of immunosuppressive protein PD-L1 in HepG2 cells after treatment using 20 μM FOXM1-PROTAC over 24 h. Results suggested that 20 μM FOXM1-PROTAC can decrease the expression of GLUT1 and PD-L1 (Fig. 6A). Subsequently, we wanted to confirm that FOXM1-PROTAC could inhibit glucose metabolism by inhibiting FOXM1. The lactate content in cells was tested to validate the effects of FIP-1 and FOXM1-PROTAC on glucose metabolism. The data indicated that the lactate concentration in cells decreased after FOXM1-PROTAC treatment, which was caused by a decreased level of glucose metabolism (Fig. 6B). We then monitored the extracellular acid of the treated cells in real-time with the hippocampus detector, and found that the secretion of extracellular acid in treated cells was reduced, which also hinted that the cell glucose metabolism ability was weakened (Fig. 6C). To assess that the decrease in glucose metabolism is due to a decrease in glucose transport, we tested the uptake of glucose analog 2-NBDG in HepG2 cells using flow cytometry. HepG2 cells cultured using glucose-free medium and 20 μM FOXM1-PROTAC, showed a lower fluorescence intensity (Fig. S5A, Supporting Information). This result was similar to the uptake of 2-NBDG that was observed by microscope (Fig. S5B, Supporting Information). We then added glucose metabolism inhibitor 2-DG to HepG2 cells and tested for the expression of PD-L1 before and after addition. It was found that the addition of 2-DG could inhibit the expression of PD-L1 (Fig. 6D). We also stained the tumor of nude mice transplanted with HepG2 cell line after treatment, and determined that FOXM1-PROTAC can also inhibit the expression of GLUT1 and PD-L1 in vivo, which is consistent with our previous in vitro research results (Fig. 6E). The results of flow cytometry announced that the PD-L1 on the membrane of HepG2 cells, treated with FIP-1 or FOXM1-PROTACs, was significantly decreased, indicating that it was peptide-dependent (Fig. 6F and Fig. S5C, Supporting Information). Separately, we performed immunofluorescence staining on the tumors of treated mice in the experimental and control groups, and determined that FOXM1-PROTAC significantly inhibited the expression of GLUT1 and PD-L1 supporting previous in vitro results (Fig. 6G).

## Discussion

The principle of PROTAC is that this bifunctional molecule binds the intracellular or nuclear targets at one end, and binds an E3 ligase at the other end, which forms a ternary complex to recruit the cellular ubiquitin proteasome system (UPS) for proteasomal degradation of targets. There are many different protein targets are successfully degraded by PROTAC, including estrogen receptor (ER), androgen receptor (AR), bromodomain-containing protein 4 (BRD4), anaplastic lymphoma kinase (ALK), et al. Currently, the small molecules are usually used as targeting warheads to design the PROTACs, and this type of PROTAC is heavily rely on the binding pockets of targets. Compared with small molecule PROTACs, the design and synthesis of p-PROTACs is simpler, and possess the ability to target “undruggable” targets with high specificity and low toxicity, and can resist to mutation targets. Since GLUT1 levels can correlate with response to FOXM1, there is increasing interest in understanding cell proliferation and glycolysis [42, 43]. However, while PD-L1 regulation has been extensively studied in cancer cells, the specific link between PD-L1 and FOXM1 has received less attention. Jingtong Zhang, et. Al., have reported that down-regulation of WDR5 will inhibit the expression of FOXM1, and further research shows that it reduced the expression of PD-L1 [44]. Hence, it is particularly momentous to explore the relationship between FOXM1 and immunosuppressive point PD-L1 from the perspective of glucose metabolism, a vital movement of cells. Also, proteolysis-targeting chimeras was used to degrade targeting protein, aiming to develop new drugs against different diseases, including cancer [45, 46].

To address unmet needs, we sought to develop a system combining the inhibitory function of peptide inhibitors with the powerful effect of protein degradation associated with proteolysis-targeting chimeras. Additionally, we attempt to clarify the relationship between cell proliferation, metabolism and immunosuppression. Through screening of a phage display library, we successfully obtained FOXM1-binding antagonistic peptide and proved that it has a strong affinity with FOXM1. We observed significant inhibition of cell viability by FIP-1, which is composed of FOXM1-targeted peptide with the addition of a cell-penetrating peptide sequence. We then selected pomalidomide as the E3 ubiquitin ligase ligand and conjugated it with FIP-1 to form a new PROTAC. This novel peptide based FOXM1-PROTAC showed two main advantages: on the one hand, the antagonistic peptide FIP-1 not only can bind with FOXM1 and recruit E3 ubiquitin to degrade FOXM1 through pomalidomide, but also can inhibit the effects of FOXM1 in cancer progression. Even there is still a little amount of FOXM1 is not degraded by FOXM1-PROTAC, the FIP-1 can also suppress the remain of FOXM1 to down-regulated the oncogenes, such as GLUT1 and PD-L1. On the other hand, the TAT used in our FOXM1-PROTAC has successfully improved the permeability of FIP-1 peptide and facilitated FOXM1-PROTAC enter cancer cells to degrade FOXM1. The biodistribution of injected FOXM1-PROTACs tells us that the p-PROTACs was a strong performance of specific targeting, and no substantial side effect was produced in “off-target” tissues.

## Conclusions

Taken altogether, we designed and synthesized a FOXM1-targeted PROTAC and investigated the relationship of FOXM1 with metabolism and immunosuppression from the perspective of cell proliferation, which gives to the advantages that small molecular antagonistic peptides could accurately bind to target proteins and Proteolysis-targeting chimeras could effectively degrade proteins. More therapeutic targets will be discovered as the biomedical field advances, and novel therapeutics will be created, especially utilizing proteolysis-targeting chimeras. FOXM1 as a key proliferation-related transcription factor, will also play a vital role in cancer treatment. However, further research is needed to uncover other vital links between FOXM1 and immunity.

## Supplementary Information

**Additional file 1: Supplementary Fig. 1.** Analysis of peptide screening results and molecular docking. A, Second round result of screening FOXM1 targeted peptides using an in vitro phage display. B, Third round result of screening FOXM1 targeted peptides using an in vitro phage display. C, Peptide characteristic analysis. d, Molecular docking of FIP-1 and FOXM1-PROTAC with Pymol. **Supplementary Fig. 2.** Western blotting results. A, MS detection of FIP-1 and FOXM1-PROTAC. B, Western blotting examination for FoxM1 of HepG2 cells treated with 20 μM FIP-1 for different time (0, 3, 6, 12, 24, 48 h). C, Western blotting test for FoxM1 of HepG2 cells treated for 24 h with increasing concentrations of FIP-1 (0, 2, 5, 10, 20, 30, 50 μM). **Supplementary Fig. 3.** FOXM1-PROTAC inhibits proliferation of HepG2 and MDA-MB-231 cells in vitro. A, The raw data of DNA content of HepG2 and MDA-MB-231cells on a Flow cytometer, treated with FIP-1 and FOXM1-PROTAC for 24 h and stained with propidium iodide. B, The examination of CDK1, CyclinB1 and CDC25B level of HepG2 and MDA-MB-231 cells, treated with FIP-1 and FOXM1-PROTAC for 48 h, using Western blotting. **Supplementary Fig. 4.** Toxicity test of FOXM1-PROTAC in vivo. A, Changes of tumor volume and statistical diagram of tumor weight. B, Changes of body weight of nude mice after caudal vein injection. C, Immunohistochemistry of Heart, Liver, Spleen, Lung and Kidney Treated by FIP-1 and FOXM1-PROTAC for 14 Days (20 mg/kg) . D, E, Activities of serum aspartic acid transferase (AST), creatinine and Blood urea nitrogen did not elevate or reduce significantly both in mice injecting with HepG2 and MDA-MB-231 cells. **Supplementary Fig. 5.** The uptake of 2-NBDG in HepG2 cells. A, The raw data of 2-NBDG fluorescence intensity in HepG2 cells on a Flow cytometer. Cells were treated with FIP-1 and FOXM1-PROTAC for 24 h and 2-NBDG for 30 min. B, The fluorescence of 2-NBDG in HepG2 cells imaged with the fluorescent Con-focal microscope. Cells were treated with FIP-1 and FOXM1-PROTAC for 24 h and 2-NBDG for 30 min. C, The PD-L1 on the membrane of HepG2 cells, treated with FIP-1 or FOXM1-PROTAC, was determined by flow cytometry.

## Data Availability

All data generated or analyzed during this study are included in this published article.

## Abbreviations

CDC-25B: Cell division cycle protein 25B
DMEM: Dulbecco’s modified eagle medium
ELISA: Enzyme-linked immunosorbent assay
FBS: Fatal bovine serun
FITC: Fluorescein isothiocyanate
FOXM1: Forkhead box M1
GLUT1: Glucose transporter-1
HPLC: High Pressure Liquid Chromatography
IPTG: Isopropyl-β-D-thiogalactoside
LB: Luriabertani
PD-L1: Programmed cell death ligand-1
PBS: Phosphate buffered saline
qPCR: Real-time quantitative PCR
PCNA: Proliferating cell nuclear antigen
PI: Propidium iodide
p-PROTACs: Peptide proteolysis-targeting chimeras
DAPI: 2-(4-Amidinophenyl)-6-indolecarbamidine dihydrochloride
