# Supplementary material for: Peptide-based PROTAC degrader of FOXM1 suppresses cancer and decreases GLUT1 and PD-L1 expression
Source: J Exp Clin Cancer Res. 2022 Sep 29;41:289. doi: 10.1186/s13046-022-02483-2 (PMC9520815; doi:10.1186/s13046-022-02483-2)
Supplement: Supplementary file 1 — Additional file 1: Supplementary Fig. 1. Analysis of peptide screening results and molecular docking. A, Second round result of screening FOXM1 targeted peptides using an in vitro phage display. B, Third round result of screening FOXM1 targeted peptides using an in vitro phage display. C, Peptide characteristic analysis. d, Molecular docking of FIP-1 and FOXM1-PROTAC with Pymol. Supplementary Fig. 2. Western blotting results. A, MS detection of FIP-1 and FOXM1-PROTAC. B, Western blotting examination for FoxM1 of HepG2 cells treated with 20 μM FIP-1 for different time (0, 3, 6, 12, 24, 48 h). C, Western blotting test for FoxM1 of HepG2 cells treated for 24 h with increasing concentrations of FIP-1 (0, 2, 5, 10, 20, 30, 50 μM). Supplementary Fig. 3. FOXM1-PROTAC inhibits proliferation of HepG2 and MDA-MB-231 cells in vitro. A, The raw data of DNA content of HepG2 and MDA-MB-231cells on a Flow cytometer, treated with FIP-1 and FOXM1-PROTAC for 24 h and stained with propidium iodide. B, The examination of CDK1, CyclinB1 and CDC25B level of HepG2 and MDA-MB-231 cells, treated with FIP-1 and FOXM1-PROTAC for 48 h, using Western blotting. Supplementary Fig. 4. Toxicity test of FOXM1-PROTAC in vivo. A, Changes of tumor volume and statistical diagram of tumor weight. B, Changes of body weight of nude mice after caudal vein injection. C, Immunohistochemistry of Heart, Liver, Spleen, Lung and Kidney Treated by FIP-1 and FOXM1-PROTAC for 14 Days (20 mg/kg) . D, E, Activities of serum aspartic acid transferase (AST), creatinine and Blood urea nitrogen did not elevate or reduce significantly both in mice injecting with HepG2 and MDA-MB-231 cells. Supplementary Fig. 5. The uptake of 2-NBDG in HepG2 cells. A, The raw data of 2-NBDG fluorescence intensity in HepG2 cells on a Flow cytometer. Cells were treated with FIP-1 and FOXM1-PROTAC for 24 h and 2-NBDG for 30 min. B, The fluorescence of 2-NBDG in HepG2 cells imaged with the fluorescent Con-focal microscope. Cells were treated wi [file 13046_2022_2483_MOESM1_ESM.docx]

**Supplementary figures and figure legends**


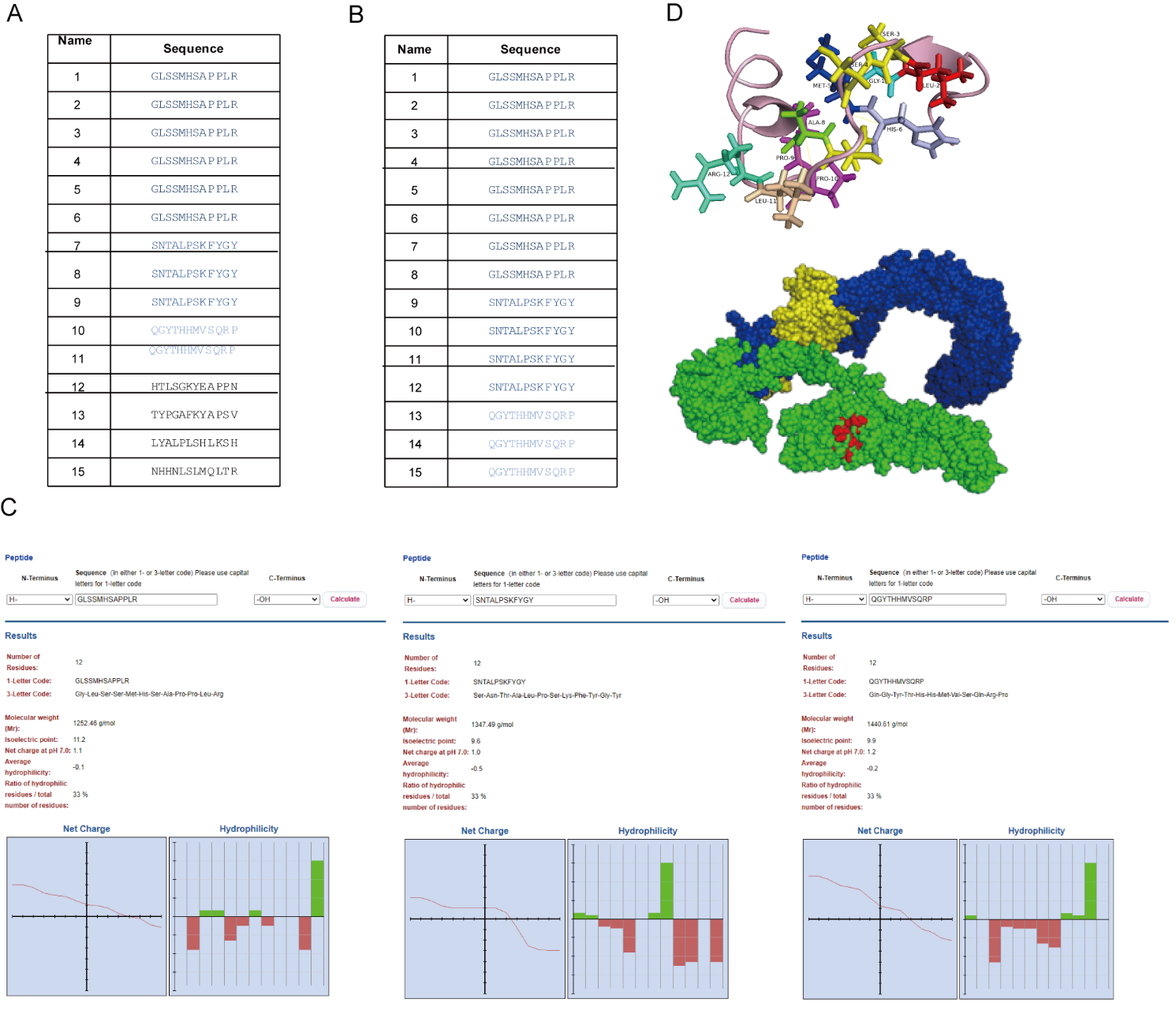


**Supplementary Fig. 1** Analysis of peptide screening results and molecular docking. A, Second round result of screening FOXM1 targeted peptides using an in vitro phage display. B, Third round result of screening FOXM1 targeted peptides using an in vitro phage display. C, Peptide characteristic analysis. d, Molecular docking of FIP-1 and FOXM1-PROTAC with Pymol.


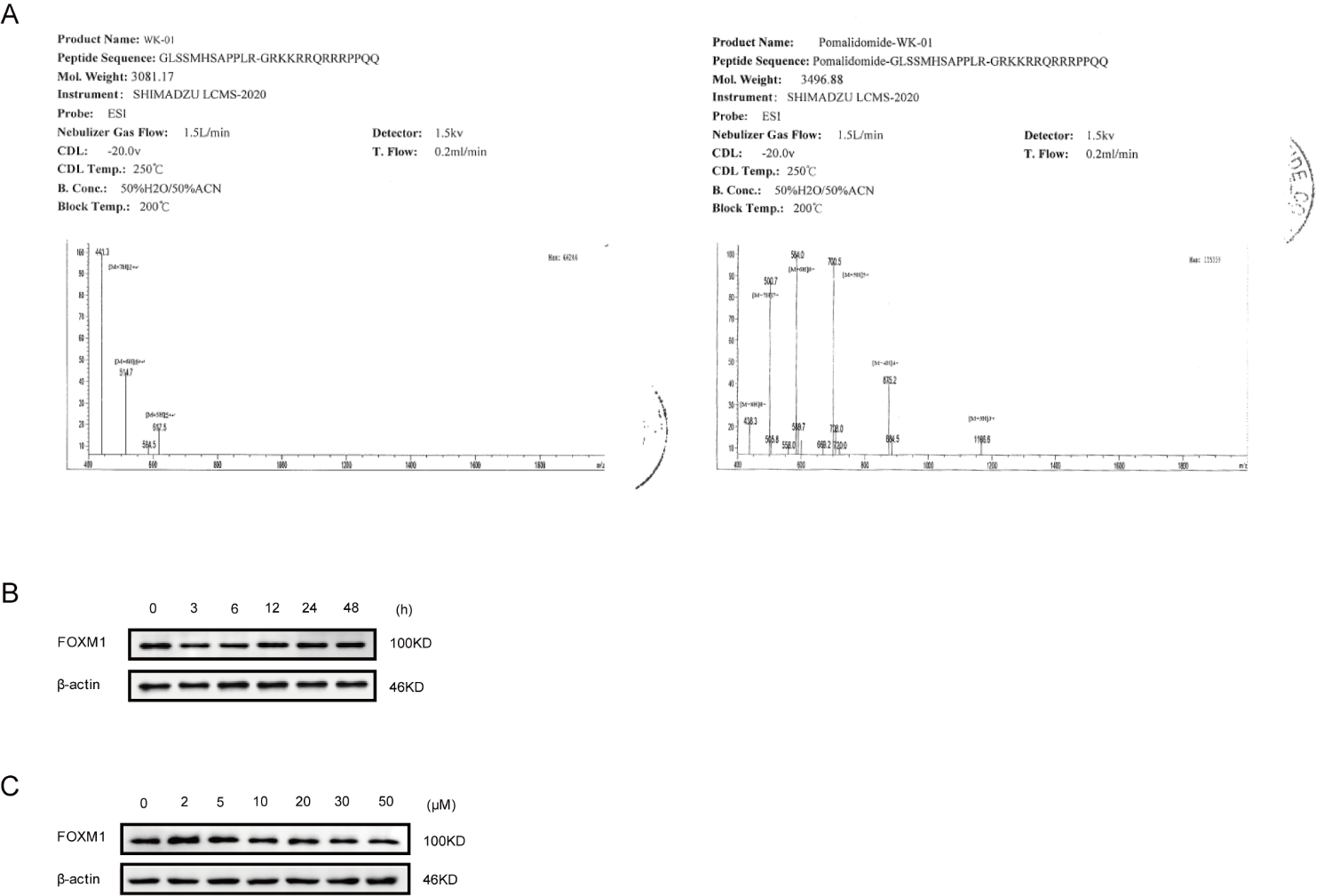


**Supplementary Fig. 2** Western blotting results. A, MS detection of FIP-1 and FOXM1-PROTAC. B, Western blotting examination for FoxM1 of HepG2 cells treated with 20 μM FIP-1 for different time (0, 3, 6, 12, 24, 48 h). C, Western blotting test for FoxM1 of HepG2 cells treated for 24 h with increasing concentrations of FIP-1 (0, 2, 5, 10, 20, 30, 50 μM).


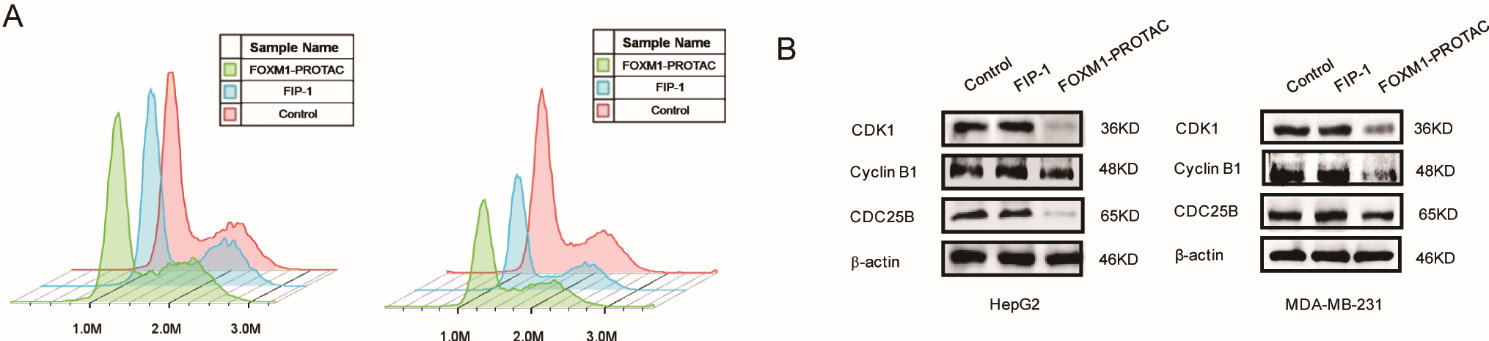


**Supplementary Fig. 3** FOXM1-PROTAC inhibits proliferation of HepG2 and MDA-MB-231 cells *in vitro*. A, The raw data of DNA content of HepG2 and MDA-MB-231cells on a Flow cytometer, treated with FIP-1 and FOXM1-PROTAC for 24 h and stained with propidium iodide. B, The examination of CDK1, CyclinB1 and CDC25B level of HepG2 and MDA-MB-231 cells, treated with FIP-1 and FOXM1-PROTAC for 48 h, using Western blotting.


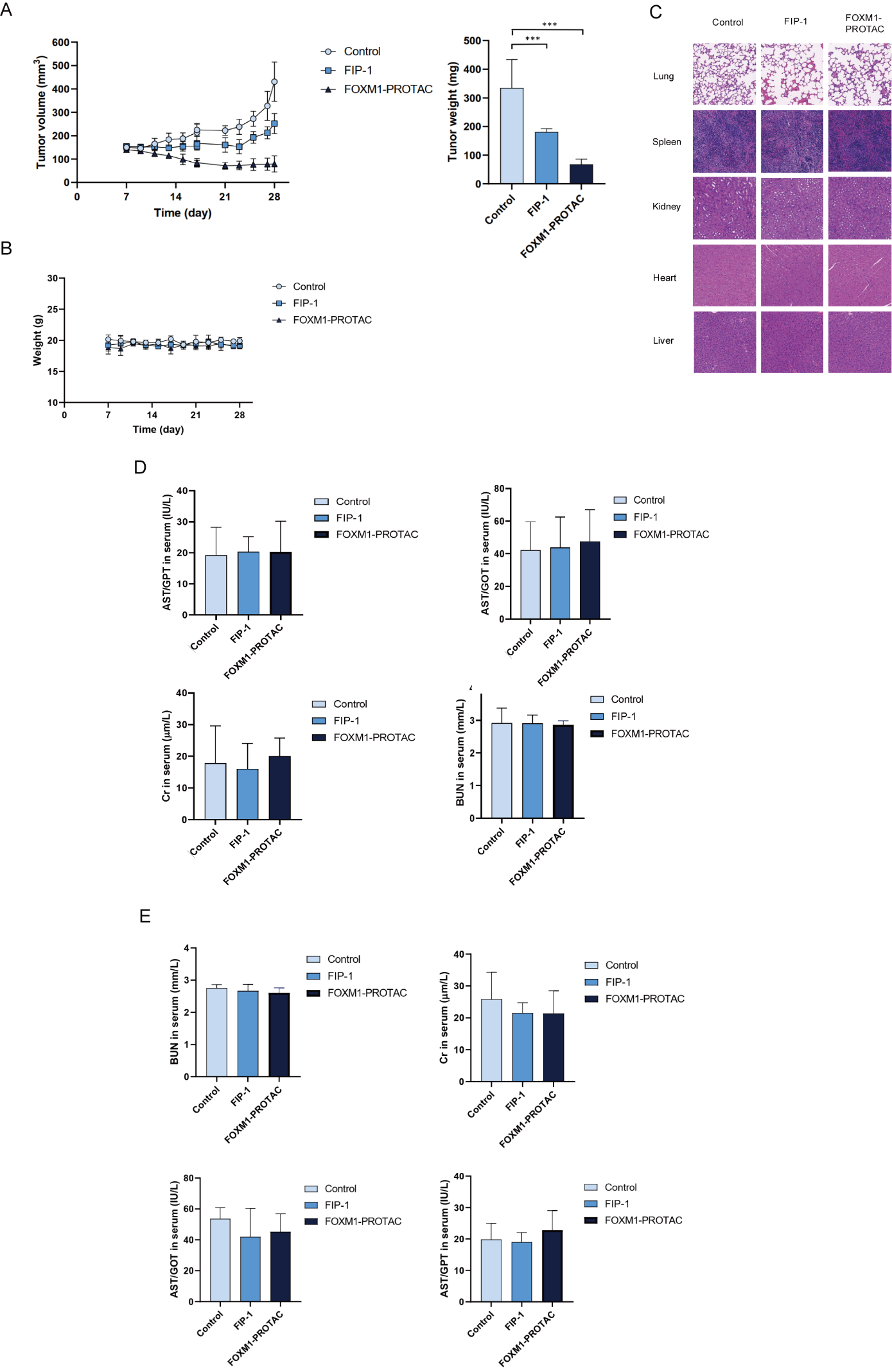


**Supplementary Fig. 4** Toxicity test of FOXM1-PROTAC *in vivo*. A, Changes of tumor volume and statistical diagram of tumor weight. B, Changes of body weight of nude mice after caudal vein injection. C, Immunohistochemistry of Heart, Liver, Spleen, Lung and Kidney Treated by FIP-1 and FOXM1-PROTAC for 14 Days (20 mg/Kg) . D, E, Activities of serum aspartic acid transferase (AST), creatinine and Blood urea nitrogen did not elevate or reduce significantly both in mice injecting with HepG2 and MDA-MB-231 cells.


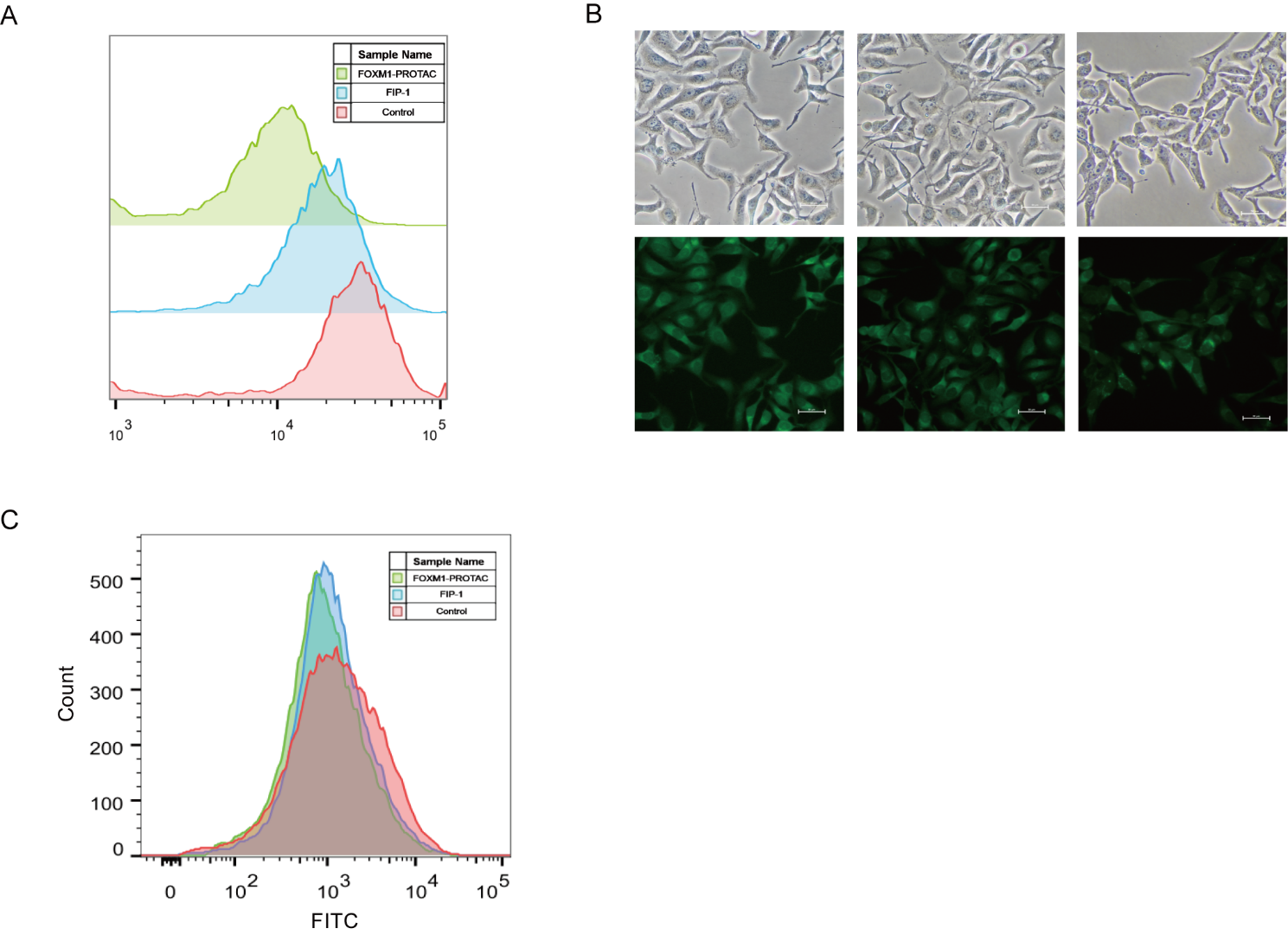


**Supplementary Fig. 5** The uptake of 2-NBDG in HepG2 cells. A, The raw data of 2-NBDG fluorescence intensity in HepG2 cells on a Flow cytometer. Cells were treated with FIP-1 and FOXM1-PROTAC for 24 h and 2-NBDG for 30 min. B, The fluorescence of 2-NBDG in HepG2 cells imaged with the fluorescent Con-focal microscope. Cells were treated with FIP-1 and FOXM1-PROTAC for 24 h and 2-NBDG for 30 min. C, The PD-L1 on the membrane of HepG2 cells, treated with FIP-1 or FOXM1-PROTAC, was determined by flow cytometry.
